# Preparation, Antioxidant Properties and Ability to Increase Intracellular NO of a New Pyridoxine Derivative B6NO

**DOI:** 10.3390/antiox10091451

**Published:** 2021-09-13

**Authors:** Anastasia Balakina, Tatyana Prikhodchenko, Vera Amozova, Tatyana Stupina, Victoria Mumyatova, Margarita Neganova, Ilya Yakushev, Alexey Kornev, Svyatoslav Gadomsky, Boris Fedorov, Denis Mishchenko

**Affiliations:** 1 Institute of Problems of Chemical Physics, RAS, 142432 Chernogolovka, Russia; t_prikhodchenko@list.ru (T.P.); amozovavi@gmail.com (V.A.); stupina.tat@gmail.com (T.S.); derevkova_viktoriya@mail.ru (V.M.); abkornev@yandex.ru (A.K.); sgadomsky@gmail.com (S.G.); ezh-77@mail.ru (B.F.); mdv@icp.ac.ru (D.M.); 2 Institute of Physiologically Active Compounds, RAS, 142432 Chernogolovka, Russia; neganova83@mail.ru; 3 Kurnakov Institute of General and Inorganic Chemistry, RAS, 119991 Moscow, Russia; ilya.yakushev@igic.ras.ru; 4 Scientific and Educational Center in Chernogolovka of Moscow Region State University, 141014 Mytishi, Russia

**Keywords:** pyridoxine, nitrogen monoxide, oxidative stress, Fenton reaction

## Abstract

In the case of various pathologies, an imbalance between ROS generation and the endogenous AOS can be observed, which leads to excessive ROS accumulation, intensification of LPO processes, and oxidative stress. For the prevention of diseases associated with oxidative stress, drugs with antioxidant activity can be used. The cytotoxic, antioxidant, and NO-donor properties of the new hybrid compound B6NO (di(3-hydroxy-4,5-bis(hydroxymethyl)-2-methylpyridinium) salt of 2-(nitrooxy)butanedioic acid) were studied. It was determined that B6NO chelates iron ions by 94%, which indicates B6NO’s ability to block the Fenton reaction. The hybrid compound B6NO inhibits the process of initiated lipid peroxidation more effectively than pyridoxine. It was shown that B6NO exhibits antioxidant properties by decreasing ROS concentration in normal cells during the oxidative stress induction by *tert*-Butyl peroxide. At the same time, the B6NO antioxidant activity on tumor cells was significantly lower. B6NO significantly increases the intracellular nitrogen monoxide accumulation and showed low cytotoxicity for normal cells (IC_50_ > 4 mM). Thus, the results indicate a high potential of the B6NO as an antioxidant compound.

## 1. Introduction

Oxidative stress plays an important role in the pathogenesis of a number of socially significant diseases, such as carcinogenesis, ischemic heart disease, atherosclerosis, and diabetes. Oxidative stress is caused by an imbalance between the antioxidant defense system (AOS) and the formation of reactive oxygen species (ROS). Excessive accumulation of ROS, which include superoxide anion radicals (O_2_^•−^), hydroxyl radicals (^•^OH), hydrogen peroxide (H_2_O_2_), and singlet oxygen (^1^O_2_), can damage biological membrane lipids, proteins, DNA, and carbohydrates [1,2].

Cancer is one of the leading causes of death in the world. The mechanism of anticancer drugs action often is based on their ability to intensify the ROS generation and initiate the processes of tumor cells free radical damage [3]. The maximum effectiveness of oncological diseases chemotherapy is achieved by using high doses of anticancer agents such as alkylating agents (cyclophosphamide), anthracycline antibiotics (doxorubicin), platinum complexes (cisplatin), and others. This makes it possible to increase the chemotherapy effectiveness for malignant neoplasms of various localizations. However, it can lead to a wide range of adverse reactions, the most dangerous of which is damage to internal organs. Toxic damage to internal organs is also based on the formation of toxic metabolites and ROS, which enhance biological membranes lipid peroxidation (LPO), leading to the cell necrosis development [4]. So, for example, the use of doxorubicin, cisplatin, and other chemotherapeutic drugs can lead to cardiotoxicity [5]. 

For the prevention of diseases associated with oxidative stress, drugs with antioxidant activity can be used [2,6,7]. It is known that compounds with both antioxidant and NO-donating properties can have a positive effect on the cardiovascular system (CVS). The use of antioxidants can reduce the side effects of anticancer chemotherapy by preventing damage to normal cells, but can reduce the effectiveness of anticancer drugs by reducing oxidative damage to tumor cells [8]. 

Vitamin B6 (also known as pyridoxine) is a coenzyme involved in over 100 metabolic reactions of amino acids, glucose, lipids, and DNA [9]. Several studies have shown that vitamin B6 may play an important role in the antioxidant defense against oxidative stress. The exact mechanism of the vitamin B6 antioxidant action is not completely clear. Nevertheless, it is known that vitamin B6 can inhibit the superoxide radicals formation [10], is an effective singlet oxygen quencher [11,12] and is able to directly react with peroxide radicals and thereby inhibit lipid peroxidation [13]. An indirect mechanism of the vitamin B6 antioxidant activity is associated with its ability to influence the conversion of homocysteine to cysteine, which plays an important role in the glutathione-dependent antioxidant defense system [14]. Thus, it can be assumed that vitamin B6 can be used to create potential drugs with pronounced antioxidant properties [15]. 

Nitrogen monoxide (NO) exhibits a wide range of physiological activities, affecting the processes of carcinogenesis [16], cell death [17], inflammation, signaling [18], and the functioning of the CVS [19]. Thus, the creation of hybrid molecules combining the antioxidant properties and a NO donating activity is very promising.

We have developed a pyridoxine-based hybrid compound (B6NO), since it is assumed that the new compound is capable of combining both antioxidant properties and NO-donating activity.

The aim of this work is to study the cytotoxic, antioxidant, and NO-donor properties of the hybrid B6NO compound.

## 2. Materials and Methods

### 2.1. Materials and Equipment

Analytical instruments. H^1^ NMR spectra were recorded on a DRX 500 spectrometer (Bruker, Billerica, MA, USA) with an operating frequency of 500.13 MHz at a temperature of 298 K in DMSO-*d*_6_. IR spectra were recorded on an ALPHA spectrometer (Bruker, Billerica, MA, USA).

Melting points were measured on a heating table BOETSIUS RN MK 05 with an observation device. Elemental analysis was performed on a CHNS/O elemental analyzer Vario Micro cube (Elementar, Germany).

Reagents and solvents. The following commercially available reagents and solvents were used in the work: pyridoxine hydrochloride (≥98%, HPLC, CAS 58-56-0, Sigma-Aldrich, Burlington, MA, USA); malic acid racemate (ReagentPlus^®^, ≥99%, CAS 6915-15-7, Sigma-Aldrich, USA), anhydrous isopropyl alcohol (anhydrous, 99.5%, Sigma-Aldrich, USA), methyl alcohol (anhydrous, 99.8%, Sigma-Aldrich, Burlington, MA, USA), nitric acid 65.54% (reagent grade), sulfuric acid (concentrated reagent grade), benzene (reagent grade), hexane (reagent grade), ethyl acetate (reagent grade), and P_2_O_5_ (reagent grade).

Single crystal X-ray diffraction studies. The X-ray diffraction data for compound B6NO were collected on the “Belok” beamline at the Kurchatov Synchrotron Radiation Source (National Research Center “Kurchatov Institute”, Moscow, Russian Federation) in *φ*-scan mode using using SX165 CCD detector (Rayonix, Evanston, IL, USA), λ = 0.74500 Å [20]. The raw data were treated with the *XDS* data reduction program [21], including absorption correction. The crystal structure was solved by direct methods [22] and refined by the full-matrix least-squares on *F*^2^ [23] using *OLEX2* structural data visualization and analysis program [24]. All non-hydrogen atoms were refined using anisotropic displacement parameters. Hydrogen atoms were located from difference Fourier maps and refined isotropically, without any constraints or restraints. Crystal data and structure refinement details are presented in Tables S1–S3. Crystallographic data for the title compound has been deposited at the Cambridge Crystallographic Data Center as supplementary publication, No 2092039 (deposit@ccdc.cam.ac.uk, http://www.ccdc.cam.ac.uk/data_request/cif, accessed date: 12 September 2021).

### 2.2. Chemistry

#### 2.2.1. Synthesis of Compound **I** 2-(Nitrooxy)butanedioic Acid

**Figure 1:**
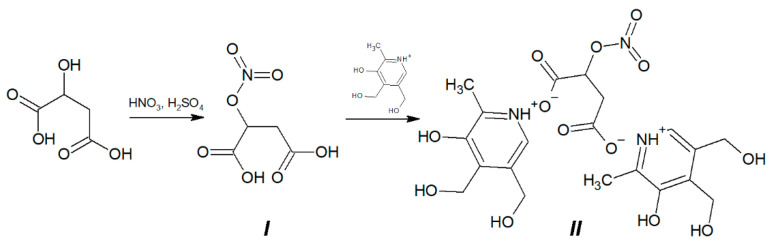
Synthesis of di(3-hydroxy-4,5-bis(hydroxymethyl)-2-methylpyridinium) salt of 2-(nitrooxy)butanedioic acid hybrid molecule B6NO (**II**) from 2-(nitrooxy)butanedioic acid (**I**).

15 g (111.9 mmol) of malic acid was added into a mixture of 30 mL of HNO_3_ and 10 mL of H_2_SO_4_ at t = 10 °C (Figure 1). Resulted suspension was being stirred for 40 min at 5 °C, then poured into ice water. The product was extracted by ethyl acetate. After removing of the solvent 12 g (the yield is 60%) of colorless crystals was obtained. M.p. 133–135 °C (decomp.). Found, %: C, 26.68; H, 2.93; N, 7.97; C_4_H_5_NO_7_. Calculated, %: C, 26.83; H, 2.81; N, 7.82. O 62.54. ^1^H NMR spectrum (DMSO-d6), δ, ppm (J, Hz): 2.90–2.95 (dd, 1H (CH_2_) J = 8.0), 2.98–3.02 (dd, 1H (CH_2_) J = 4.0), 5.63–5.65 (m, 1H (CH), J1 = 4.0, J2 = 8.0), 12.0–14.0 (broad singlet, HOOC). IR spectrum (cm^−1^): 1732; 1722; 1654; 1444; 1385; 1336; 1273; 1249; 1205; 1053; 907; 844; 751; 673.

#### 2.2.2. Synthesis of Compound **II** Di(3-hydroxy-4,5-bis(hydroxymethyl)-2-methylpyridinium) Salt of 2-(Nitrooxy)butanedioic Acid

0.42 g of 2-(nitrooxy)butanedioic acid **I** (2.35 mmol) was added into suspension of 0.8 g (4.7 mmol) pyridoxine in 30 mL of anhydrous methanol (Figure 1). The reaction mixture was stirred for 2 h at 20 °C then was cooled to −15 °C for 1 h. The resulted precipitate was filtered of and washed by ethyl acetate. 1.09 g of colorless crystals was obtained (the yield is 89.34%). 

M.p. 110–112 °C (with decomposition). Found, %: C, 46.92; H, 5.39; N, 7.80. C_4_H_5_NO_7_. Calculated, %: C, 46.42; H, 5.26; N, 8.12; O 40.20. ^1^H NMR spectrum (DMSO-d6), δ, ppm (J, Hz): 2.38 (s, 6H, -CH_3_), 2.8–3.0 (m, 2H, CH_2_), 3.0–4.3 (broad singlet, HO-), 4.53 (s, 4H, CH_2_-OH), 4.74 (s, 4H, CH_2_-OH), 5.54–5.56 (m, 1H, -CH-), 7.93 (s, 2H, Ar-H). IR spectrum (cm^−1^): 1622; 1590; 1544; 1382; 1343; 1275; 1090; 1032; 904; 861; 743.

NMR and IR spectra of compounds **I** (NMA) and **II** (B6NO) are presented in Supplementary Materials (Figures S2–S4).

### 2.3. Biology

Mammalian cells. The following cell cultures were used in the work: HepG2 (human hepatocellular carcinoma), Vero (African green monkey kidney epithelial cells). All cell cultures were obtained from the collection of the Institute of Cytology of the Russian Academy of Sciences.

Mammalian cell culture. Cultivation of the cells was carried out according to a conventional procedure in an atmosphere of 5% CO_2_ and a temperature of 37 °C. Vero cells were cultured in DMEM (PanEco, Russia) supplemented with 10% fetal bovine serum (BioWest, France), penicillin (50 U·mL^−1^), and streptomycin (50 mg·mL^−1^). HepG2 cells were cultured in EMEM medium (PanEco, Russia) with the addition of 10% fetal bovine serum, penicillin (50 U·mL^−1^), and streptomycin (50 mg·mL^−1^). 

Cytotoxicity. The cytotoxic properties of the B6 vitamin and B6NO were studied using the MTT test. All cells were plated in culture 96-well plates at a concentration of 5 × 10^4^ cells mL^−1^. The compounds were introduced into the culture medium after 24 h of cultivation. The dye 3-(4,5-dimethylthiazol-2-yl)-2,5-diphenyl-2H-tetrazolium bromide (MTT, DiaM, Russia) was added to the incubation medium after 24 h of the test complex administration at a concentration of 0.5 mg·mL^−1^. The formed formazan crystals were dissolved in 100% DMSO. Optical density measurement was carried out at a main wavelength of 570 nm and a background wavelength of 620 nm using a multifunctional microplate reader Spark 10M (Tecan, Switzerland). The cytotoxicity index (IC_50_) was determined on the basis of dose-dependent curves using a median effect analysis by the Chou T.C. and Talalay P. method [25].

Intracellular ROS accumulation. The study was carried out using a fluorescent dye DCFH-DA (Sigma-Aldrich, Burlington, MA, USA) as described earlier [26]. All cells were plated into culture 96-well plates at a concentration 2.5 × 10^5^ cells per well. The compound under study was introduced into the culture medium 24 h after sieving. The culture medium was removed after 24 h, the cells were washed with PBS buffer (pH 7.4), then incubated for 30 min in an atmosphere of 5% CO_2_ and 37 °C with DCFH-DA dye at a concentration of 25 μM. Then the cells were washed with PBS buffer and incubated for 30 min in the presence of an oxidative stress inducer *tert*-Butyl peroxide (Luperox^®^, Sigma-Aldrich, Burlington, MA, USA) at a concentration of 25 μM. The fluorescence intensity was measured using a multifunctional reader Spark 10M (Tecan, Switzerland) (Ex/Em = 485/535 nm).

Intracellular NO accumulation. The cells were plated in 96-well plates in the standard incubation medium (2.5 × 10^5^ cells per well). 24 h after plating cells were washed with PBS (pH 7.4) and incubated with 5 μM DAF-FM DA (Sigma-Aldrich, Burlington, MA, USA) in PBS for 30 min at 37 °C and 5% CO_2_ [27]. After the incubation, cells were washed with PBS and the compounds under study were added at the concentration of 320 μM for B6NO and NMA, 640 μM for vitamin B6. Fluorescence intensity was detected on a plate reader Spark10M (Tecan, Switzerland) (Ex/Em = 495/515 nm).

Lipid peroxidation by MDA estimate. Male nonlinear outbred rats were used for the experiments. The animals were kept in a standard vivarium with a 12-h light regime and free access to water and food. All manipulations with animals were carried out in accordance with the decisions of the IPAC RAS Bioethics Commission. The decapitation of animals anesthetized in advance with CO_2_ was performed using a guillotine (OpenScience, Russia). The brain was homogenized at 4 °C in a buffer containing 120 mM KCl and 20 mM Hepes (pH 7.2). The quantitative determination of protein was carried out according to the standard technique using the microbiuret method [28]. To study the effect of compounds on the process of rat brain homogenate lipid peroxidation (LPO), we used a modified version of the TBA test [29], which is based on the reaction of 2-thiobarbituric acid with the final LPO product-malondialdehyde (MDA). For Fe(II)-induced LPO the samples contained the test compounds, rat brain homogenate (2.5 mg·mL^−1^), as well as 500 μM ferrous iron ions (FeSO_4_ × 10 H_2_O). After the incubation for 120 min at 37 °C for spontaneous LPO and at 30 min for Fe(II)-induced LPO, TBA reagent (0.45% thiobarbituric acid (TBA, Sigma-Aldrich, Burlington, MA, USA) and 20% TCA (Sigma-Aldrich, Burlington, MA, USA)) was added to all samples, followed by incubation for 90 min at 90 °C, and centrifugation at 6000 rpm for 15 min. Optical density measurements were carried out on an EnVision plate reader (Perkin Elmer, Waltham, MA, USA) at 540 nm.

Fe(II)-chelating activity. Fe(II)-chelating activity was determined according to the modified method described in [30]. The test compounds at a concentration of 30 μM were dissolved in ethanol and incubated for 5 min at room temperature in the presence of 500 μM FeSO_4_. Then, ferrozine was added to the mixture to a final concentration of 250 μM. After 10 minutes, the optical density was measured with an EnVision plate reader (Perkin Elmer, Waltham, MA, USA) at 562 nm.

Statistical analysis. The results of three independent experiments are presented as mean ± SD. The significance of the differences between the groups was assessed using the Student’s *t*-test. Values of *p* < 0.05 were considered statistically significant. Data were statistically processed using GraphPad software.

## 3. Results

### 3.1. The Ability of B6NO to Inhibit the Fenton Reaction

The effect of the hybrid molecule B6NO and vitamin B6 on the process of lipid peroxidation was determined by the level of the final lipid peroxidation product-malondialdehyde (MDA) using a modified TBA test. The experiment was carried out in two versions: spontaneous and initiated LPO. LPO was induced in the rat brain homogenate using the Fenton reaction in the presence of Fe^2+^ ions. 

**Figure 2:**
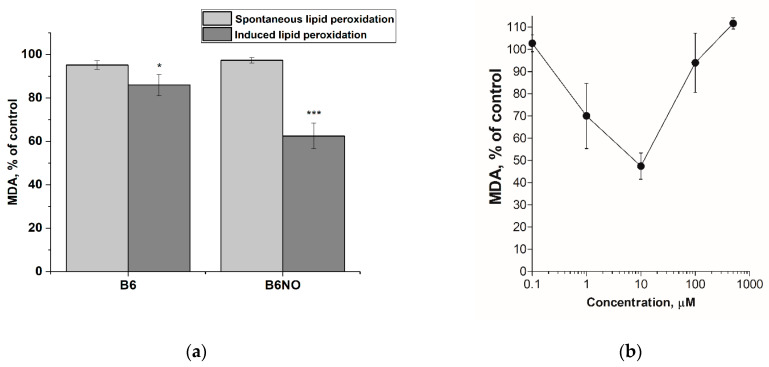
(**a**) Influence of hybrid molecule B6NO and vitamin B6 at a concentration of 30 μM on the spontaneous and iron-initiated lipid peroxidation process of rat brain homogenate. The concentration of Fe(II) was 500 μM. Significant differences are shown as * *p* < 0.05; *** *p* < 0.001. (**b**) Concentration dependence of the B6NO effect on iron-initiated lipid peroxidation.

It was shown that both compounds did not affect the processes of spontaneous lipid peroxidation in the concentration range from 1 μM to 500 μM. However, upon initiation of LPO with Fe(II), the studied compounds reduce the level of malondialdehyde in the system. The hybrid compound B6NO at a concentration of 30 μM showed pronounced antioxidant properties, inhibiting lipid peroxidation by 37.57 ± 5.92% relative to the control, while vitamin B6 at the same concentration showed insignificant activity (14.04 ± 4.94% of the control) (Figure 2a). Additionally, the concentration dependence of the effect on the MDA formation during Fe(II)-induced LPO was analyzed for the B6NO hybrid molecule in a wide concentration range (from 1 μM to 500 μM). An interesting fact was the discovery of a bell-shaped antioxidant activity dependence. Thus, the maximum effect of LPO inhibition was in the range from 5 μM to 50 μM, and at lower and higher concentrations, the effect was not observed (Figure 2b). The data obtained suggest that the antioxidant properties of B6NO are manifested precisely upon initiation of lipid peroxidation by Fe^2+^ ions, which, apparently, is associated with a certain mechanism of the compound’s action on the Fenton reaction.

**Figure 3:**
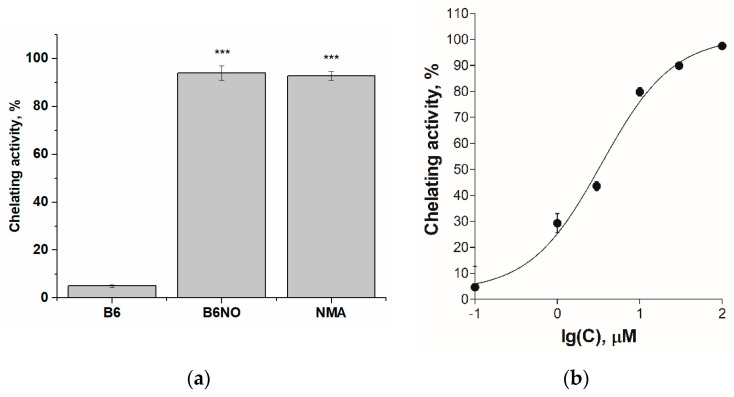
(**a**) Fe(II)-chelating activity of the B_6_, B6NO and nitromalic acid (NMA) at a concentration of 30 μM. Significant differences are shown as *** *p* < 0.001.; (**b**) Concentration dependence of the increase in Fe(II)-chelating activity of the B6NO hybrid molecule.

To confirm this assumption, studies of Fe(II)-chelating activity of vitamin B6, B6NO and nitromalic acid (NMA) were carried out. It was shown that B6NO and NMA chelates iron ions by 94% relative to the control, while vitamin B6 in equimolar concentration bound ferrous ions by no more than 10% (Figure 3a). The EC_50_ value of the chelating effect for the B6NO hybrid molecule was also determined, which was 3.44 ± 0.55 μM (Figure 3b). Obviously, the discovered B6NO ability to chelate Fe^2+^ ions leads to inhibition of the Fenton reaction, as a result of which the formation of a highly toxic hydroxyl radical (^•^OH) decreases and, as a consequence, the oxidative stress is suppressed. 

Thus, the modification of pyridoxine with nitromalic acid enhances the antioxidant properties of the B6NO hybrid molecule and also imparts iron-chelating properties to it.

### 3.2. Influence of B6NO on the Mammalian Cells Viability 

**Figure 4:**
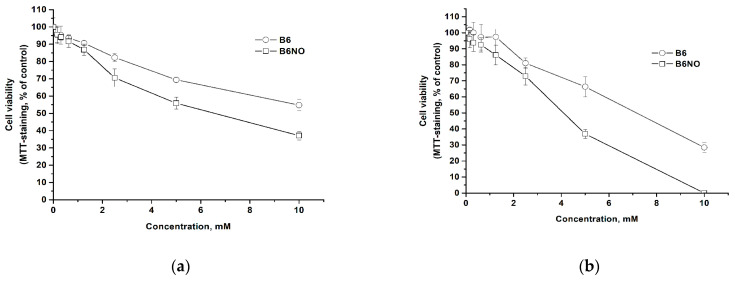
Dose-response curves for normal Vero (**a**) and cancer HepG2 (**b**) cells after 24 h of B6NO and vitamin B6 exposure.

Since B6NO is a vitamin derivative, it is expected to have low cytotoxicity. The influence on the normal and tumor cells viability was carried out using the MTT test method 24 h after the action of the test compounds. The results are shown in the Figure 4. 

It has been determined that both the hybrid compound and pyridoxine have low cytotoxicity. It should be noted that B6NO has a slightly higher cytotoxicity than the vitamin, but both compounds can be classified as low-toxic substances for cells according to the Halle & Göres classification [31]. Comparison of IC_50_ values for normal and tumor cells showed that both B6NO and pyridoxine exhibit a greater toxic effect on tumor cells as compared to normal cells (Table 1).

Thus, the new hybrid compound is low-toxic and its biological activity can be studied in cell models.

### 3.3. Antioxidant Activity of B6NO under Conditions of Oxidative Stress Induction in Normal and Tumor Cells

The study of the antioxidant properties of B6NO was carried out under oxidative stress induction by *tert*-Butyl peroxide (Luperox^®^) using a fluorescent dye dichlorofluorescein diacetate (DCFH-DA), which shows the intracellular content of ROS.

**Figure 5:**
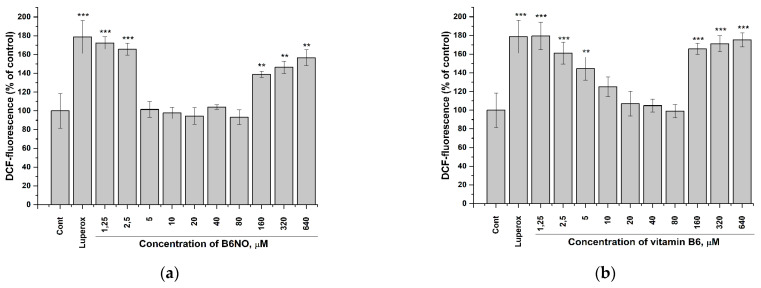
Influence of B6NO and vitamin B6 on the intracellular ROS formation initiated by *tert*-Butyl peroxide in Vero cells and measured using the DCFH-DA assay. Cells were treated with 25 µM *tert*-Butyl peroxide (Luperox^®^) for 30 min and pre-treatment with (**a**) B6NO or (**b**) vitamin B6 at 1.25−640 µM for 24 h. Significant differences are shown as ** *p* < 0.01; *** *p* < 0.001.

The hybrid compound B6NO was found to exhibits antioxidant properties in the concentration range from 5 to 80 μM, while reducing the ROS content to the control level in normal Vero cells. In addition, the molecule reduced the intracellular ROS content more effectively than pyridoxine. The results are shown in the Figure 5. 

**Figure 6:**
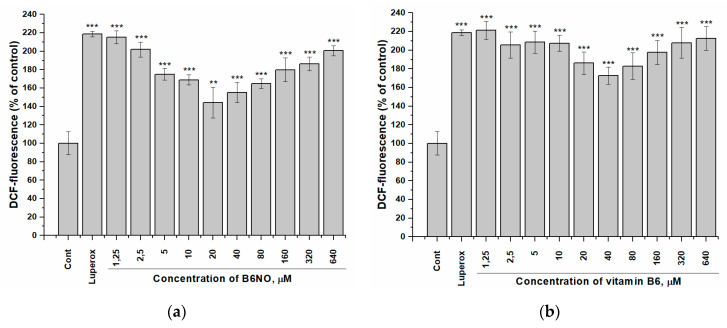
Influence of B6NO and vitamin B6 on the intracellular ROS formation initiated by *tert*-Butyl peroxide in HepG2 cells and measured using the DCFH-DA assay. Cells were treated with 25 µM *tert*-Butyl peroxide (Luperox^®^) for 30 min and pre-treatment with (**a**) B6NO or (**b**) vitamin B6 at 1.25−640 µM for 24 h. Significant differences are shown as ** *p* < 0.01; *** *p* < 0.001.

It should be noted that both B6NO and B_6_ showed a pronounced antioxidant activity only on normal Vero cells, while this effect was significantly weaker on HepG2 tumor cells (Figure 6), which indicates the selectivity action of these antioxidants.

In addition, the obtained dependences of the B6NO antioxidant effect on its concentration upon induction of oxidative stress by *tert*-Butyl peroxide have a bell-shaped appearance, which is consistent with the data on the brain homogenate model (Figure 2b).

Thus, the new hybrid compound has a high antioxidant activity during the induction of oxidative stress by both iron ions and organic peroxides.

### 3.4. NO-Donor Properties of B6NO in Normal and Tumor Cells 

**Figure 7:**
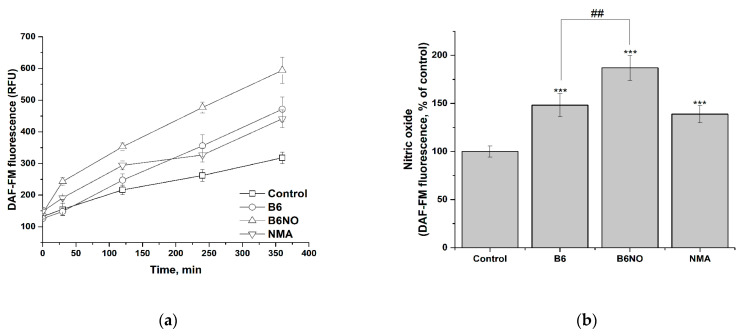
Influence of B6NO, vitamin B6 and NMA on the intracellular NO formation in Vero cells and measured using the DAF-FM assay. Time dependence of intracellular nitrogen monoxide concentration (**a**) and relative concentration of intracellular nitrogen monoxide after 6 h of exposure to compounds (**b**). Significant differences are shown as *** *p* < 0.001 vs. control; as ^##^ *p* < 0.01 B6NO vs. vitamin B6.

**Figure 8:**
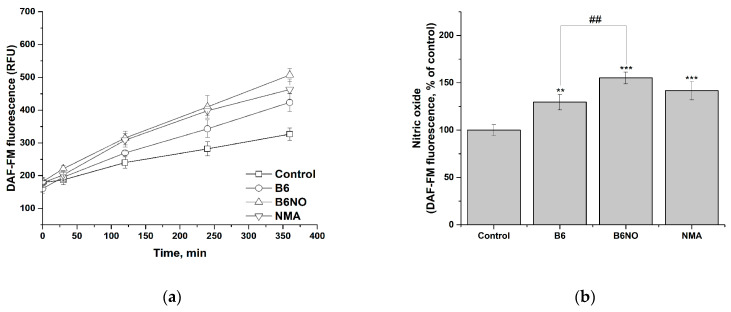
Influence of B6NO, vitamin B6 and NMA on the intracellular NO formation in HepG2 cells and measured using the DAF-FM assay. Time dependence of intracellular nitrogen monoxide concentration (**a**) and relative concentration of intracellular nitrogen monoxide after 6 h of exposure to compounds (**b**). Significant differences are shown as ** *p* < 0.01; *** *p* < 0.001 vs. control; as ^##^ *p* < 0.01 B6NO vs. vitamin B6.

The study of the nitrogen monoxide formation and intracellular accumulation under the action of B6NO was carried out using DAF-FM assay. In addition, the effect on the intracellular NO accumulation of the vitamin B6 and nitromalic acid, which are part of the hybrid molecule, was determined. It was shown that all of the studied compounds induced intracellular accumulation of nitric oxide, both in normal and tumor cells (Figure 7a and Figure 8a). 

The data obtained indicate that in both investigated cell lines, activation of endothelium NO synthase occurs under the action of the vitamin B6, which leads to a significant NO accumulation. At the same time, the contribution of the two active components of the hybrid molecule to the NO accumulation in the case of normal and tumor cells is different, which can be explained by differences in their physiological status. Thus, in the case of normal Vero cells (Figure 7b), the content of NO is determined both by the contribution of the vitamin B6 and by the biotransformation of nitromalic acid, while in tumor HepG2 cells the effect of B6NO and nitromalic acid does not differ significantly (Figure 8b).

Thereby, the accumulation of intracellular NO under the action of B6NO can occur both due to direct biotransformation of NMA nitro groups, and an increase in the availability of L-arginine for NO synthesis through endothelial NO synthase by pyridoxine.

## 4. Discussion

During normal physiological processes, there is a balance between the formation of ROS and their inactivation by the AOS. In the case of various pathologies, an imbalance between the ROS generation and the endogenous AOS can be observed, which leads to excessive ROS accumulation, intensification of LPO processes, and oxidative stress. Recently, there has been a significant increase in interest in the search for natural antioxidants for creating drugs in order to replace synthetic antioxidants, the use of which is limited due to their side effects [32,33,34,35].

A number of pathological conditions are largely associated with the lack of nitric oxide. This applies to cardiovascular, infectious, inflammatory diseases, thrombosis, malignant tumors, diseases of the genitourinary system, brain damage during strokes, etc. A literature search did not confirm a direct pathway of NO generation via vitamin B6. However, it has been shown that vitamin B6 is directly involved in the processes of NO generation by the enzyme eNOS from the amino acid L-arginine. Dysfunction of eNOS is caused by decreased availability of arginine and is accompanied by elevated homocysteine levels. In addition, the conversion of methionine to homocysteine is associated with the activation of arginine protein methyltransferases, a group of enzymes responsible for the methylation of L-arginine and the subsequent formation of asymmetric dimethylarginine (ADMA), which is a known endogenous inhibitor of eNOS. Vitamin B6 acts as an important cofactor in the conversion of homocysteine to cysteine, thereby preventing the formation of ADMA and increasing the generation of NO. It is also known that increased production of reactive oxygen species (ROS) may cause an increase in ADMA levels [36]. Therefore, the antioxidant effect of vitamin B6 may also serve as evidence of restoration of eNOS function, which may lead to an increase in NO accumulation in cells.

In addition to pyridoxine, the hybrid compound B6NO contains a nitrate group. Nitrates are the most famous class of organic compounds, the NO-donor properties of which determine their use as pharmaceuticals. It was revealed that the formation of NO from nitrates can be both enzymatic and without the participation of the enzymes catalytic effect. It is assumed that intracellular thiols are involved in the non-enzymatic pathway of NO formation from nitrates (both in vitro and in vivo) [37], and cytochrome P-450 and glutathione-S-transferase are included in the enzymatic biotransformation of organic nitrates [38,39].

We have proposed a hybrid molecule B6NO that includes a vitamin B6 and nitromalic acid. Vitamin B6 is a natural compound with pronounced antioxidant properties and the ability to influence the synthesis of nitrogen monoxide. Nitromalic acid is also a nitro derivative of natural malic acid. The hybrid compound is capable of reducing the oxidative processes development induced by organic peroxides and inhibiting the Fenton reaction by chelating metal ions with variable valence.

**Figure 9:**
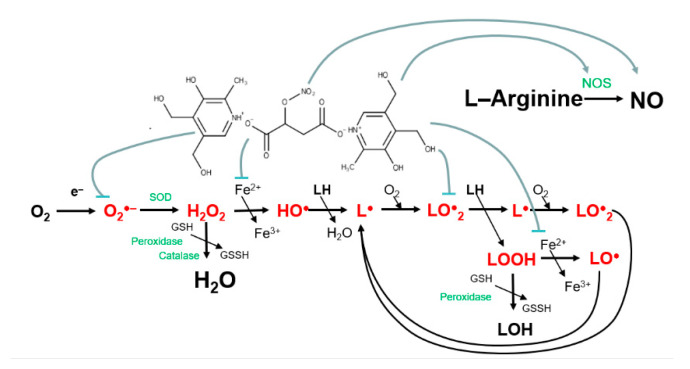
Scheme of the possible mechanism of action of the hybrid compound B6NO.

A generalized scheme of the proposed mechanism of the hybrid molecule B6NO action on oxidative processes and the intracellular nitrogen monoxide formation is shown in Figure 9.

## 5. Conclusions

The new hybrid compound B6NO has high antioxidant activity in the induction of oxidative stress by both iron ions and organic peroxides, as well as low cytotoxicity. The modification of pyridoxine with nitromalic acid enhances the antioxidant properties of the B6NO hybrid molecule, and gives it Fe(II)-chelating activity and the ability to inhibit the Fenton reaction.

The accumulation of intracellular NO under the action of B6NO can occur both due to direct biotransformation of NMA nitro groups and due to an increase in the availability of L-arginine for NO synthesis through endothelial NO-synthase under the action of pyridoxine. Thus, both pyridoxine and NMA contribute to the NO-donor properties of the hybrid molecule, which in combination with antioxidant activity can optimize the processes of intracellular synthesis of nitrogen monoxide. The results obtained show the high potential of B6NO as an antioxidant compound.

## 6. Patents

RU 2712914 C2 Mishchenko Denis V., Gadomskij Svyatoslav Ya., Yarmolenko Andrej I., Eremeev Anatolij B., Balakina Anastasiya A., Neganova Margarita E., Areshidze David A. Bis-(4,5-oxymethyl-2-methyl-3-oxy)pyridinium salt of 2-nitroxybutane-1,4-diiodic acid and method for production thereof.

## Figures and Tables

**Table 1:** Dose IC_50_ values for normal and tumor cells after 24 h of exposure to B6NO and vitamin B6.

Cells	IC_50_, mM	
	B6NO	B_6_
Vero	6.55 ± 0.76	>10
HepG2	4.70 ± 0.83	6.32 ± 0.98

## Data Availability

The authors confirm that the data supporting the findings of this study are available within the article or its Supplementary Materials.

## Supplementary Materials

The following are available online at https://www.mdpi.com/article/10.3390/antiox10091451/s1. Figure S1: Molecular and crystal structure of di(3-hydroxy-4,5-bis(hydroxymethyl)-2-methylpyridinium) salt of 2-(nitrooxy)butanedioic acid (B6NO) according to single crystal XRD experiment; Table S1: Experimental data and structure refinement for B6NO; Table S2: Hydrogen bonds for co-crystallizate B6NO; Table S3: Main bond lengths for B6NO; Figure S2: (a) IR spectrum of the compound **I** (2-(nitrooxy)butanedioic acid); (b) IR spectrum of the compound **II** (di(3-hydroxy-4,5-bis(hydroxymethyl)-2-methylpyridinium) 2-(nitrooxy)butanedioate) (KBr pellet); Figure S3: 1H NMR spectrum of compound **I** (2-(nitrooxy)butanedioic acid) (DMSO-d6); Figure S4: 1H NMR spectrum of compound **II** (di(3-hydroxy-4,5-bis(hydroxymethyl)-2-methylpyridinium) 2-(nitrooxy)butanedioate) (DMSO-d6).
