# Supplementary material for: Preparation, Antioxidant Properties and Ability to Increase Intracellular NO of a New Pyridoxine Derivative B6NO
Source: Antioxidants (Basel). 2021 Sep 13;10(9):1451. doi: 10.3390/antiox10091451 (PMC8465670; doi:10.3390/antiox10091451)
Supplement: Supplementary file 1 [file antioxidants-10-01451-s001.zip › antioxidants-1363698-supplementary.pdf]

antioxidants

MDPI

Supplementary Information

Preparation, Antioxidant Properties and Ability to Increase Intracellular NO of a New Pyridoxine Derivative B6NO

Anastasia Balakina [1,*], Tatyana Prikhodchenko [1], Vera Amozova [1], Tatyana Stupina [1], Victoria Mumyatova [1], Margarita Neganova [2], Ilya Yakushev [3], Alexey Kornev [1], Svyatoslav Gadomsky [1], Boris Fedorov, [1] and Denis Mishchenko [1,4]

[1] Institute of Problems of Chemical Physics RAS, 142432 Chernogolovka, Russia; t_prikhodchenko@list.ru (T.P.); amozovavi@gmail.com (V.A.), stupina.tat@gmail.com (T.S.); derevkova_viktoriya@mail.ru (V.M.); abkornev@yandex.ru (A.K.); sgadomsky@gmail.com (S.G.); ezh-77@mail.ru (B.F.); mdv@icp.ac.ru (D.M.)

[2] Institute of Physiologically Active Compounds RAS, 142432 Chernogolovka, Russia; neganova83@mail.ru

[3] Kurnakov Institute of General and Inorganic Chemistry RAS, 119991 Moscow, Russia; ilya.yakushev@igic.ras.ru

[4] Scientific and Educational Center in Chernogolovka of Moscow Region State University, 141014 Mytishi, Russia

* Correspondence: balakina@icp.ac.ru

Citation: Balakina, A.; Prikhodchenko, T.; Amozova, V.; Stupina, T.; Mumyatova, V.; Neganova, M.; Yakushev, I.; Kornev, A.; Gadomsky, S.; Fedorov, B.; et al. Preparation, Antioxidant Properties and Ability to Increase Intracellular NO of a New Pyridoxine Derivative B6NO. *Antioxidants* **2021**, *10*, 1451. https://doi.org/10.3390/antiox10091451

Academic Editor: Bulent Mutus

Received: 16 August 2021
Accepted: 9 September 2021
Published: 13 September 2021

Publisher's Note: MDPI stays neutral with regard to jurisdictional claims in published maps and institutional affiliations.

Copyright: © 2021 by the authors. Licensee MDPI, Basel, Switzerland. This article is an open access article distributed under the terms and conditions of the Creative Commons Attribution (CC BY) license (http://creativecommons.org/licenses/by/4.0/).

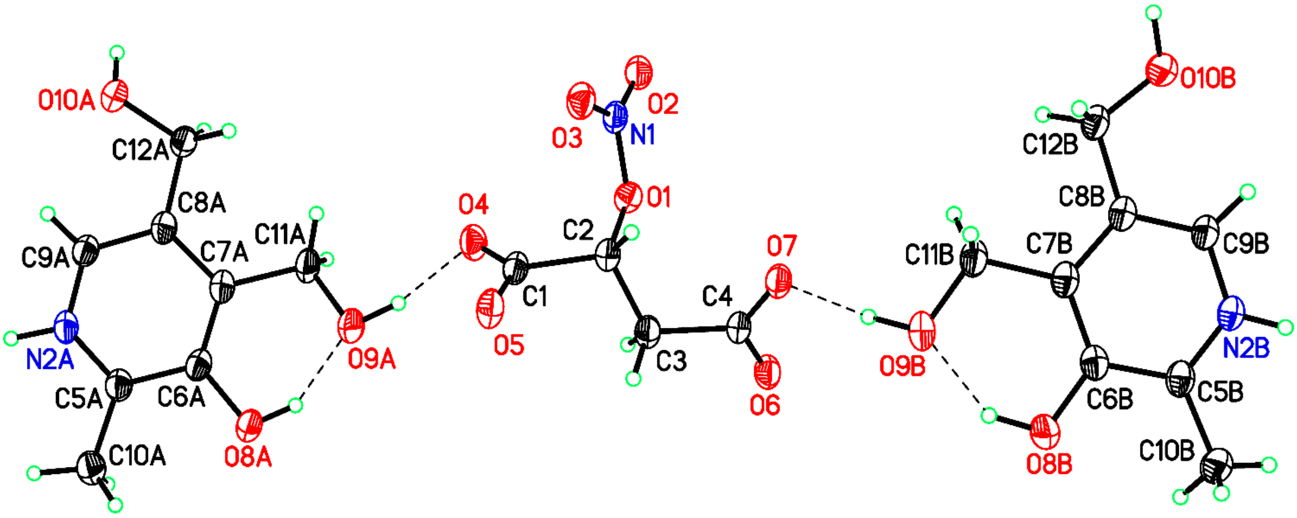

Figure S1. Molecular and crystal structure of di(3-hydroxy-4,5-bis(hydroxymethyl)-2-methylpyridinium) 2-(nitrooxy)butanedioate (B6NO) according to single crystal XRD experiment. The anisotropic displacement parameters are shown at the 50% probability level.

Table S1. Experimental data and structure refinement for B6NO.

**Crystal data**	**B6NO**
CCDC number	2092039
Empirical formula	$C_{20}H_{27}N_3O_{13}$
Formula weight	517.44
Temperature, K	100(2)
Crystal colour, habit	colorless, nugget
Crystal size, mm	0.070 × 0.060 × 0.040
Wavelength, Å	0.74500
Crystal system	Triclinic
Space group	*P*-1
a, Å	9.0792(13)
b, Å	9.7350(11)
c, Å	12.8861(15)
α, deg.	89.542(3)
β, deg.	84.801(11)
γ, deg.	78.395(11)
V, Å^3	1111.0(2)
Z	2
Density (calc.), g/cm^3	1.547
μ, mm^{-1}	0.146
F(000)	544
Theta range, deg.	1.663 – 31.003
Index ranges	$-12 \leq h \leq 12$
	$-13 \leq k \leq 13$
	$-17 \leq l \leq 17$
Reflections collected	18585
Independent reflections (R_{int})	6102 (0.0685)
R_1 / wR_2 ($I > 2\sigma(I)$)	0.0598 / 0.1426
R_1 / wR_2 (all data)	0.1071 / 0.1693
Data / restraints / parameters	6102 / 0 / 433

Goodness-of-fit on F^2	1.008
T_{min} / T_{max}	0.005 / 1.000
$\Delta\rho_{max}$ / $\Delta\rho_{min}$, e·Å^{-3}	0.389 / -0.334

Table S2. Hydrogen bonds for co-crystallizate B6NO (Å and °).

D-H...A	d(D-H)	d(H...A)	d(D...A)	<(DHA)
O(8A)-H(8A)...O(9A)	0.88(5)	1.76(4)	2.542(3)	148(4)
O(8B)-H(8B)...O(9B)	0.93(5)	1.71(5)	2.527(3)	145(4)
O(9A)-H(9A)...O(4)	0.94(4)	1.72(4)	2.648(3)	168(4)
O(9B)-H(9B)...O(7)	0.97(4)	1.68(5)	2.644(3)	172(4)
O(10A)-H(10E)...O(4)$^{\#1}$	0.87(4)	1.81(4)	2.684(3)	177(4)
O(10B)-H(10B)...O(7)$^{\#2}$	0.98(4)	1.84(4)	2.802(3)	166(4)
N(2A)-H(2A)...O(6)$^{\#3}$	0.97(4)	1.66(4)	2.629(3)	172(3)
N(2B)-H(2B)...O(5)$^{\#4}$	0.93(4)	1.83(4)	2.743(3)	169(3)

Symmetry transformations used to generate equivalent atoms:
#1 -x, -y, -z+1
#2 -x+1, -y+2, -z+1
#3 x-1, y-1, z
#4 x+1, y+1, z

Table S3. Main bond lenghts for B6NO.

Bond	Distance, Å
O(1)-N(1)	1.396(3)
O(1)-C(2)	1.451(3)
O(2)-N(1)	1.206(3)
O(3)-N(1)	1.212(3)
O(4)-C(1)	1.265(3)
O(5)-C(1)	1.248(3)
O(6)-C(4)	1.250(3)
O(7)-C(4)	1.266(3)
C(1)-C(2)	1.536(3)
C(2)-C(3)	1.517(3)
C(3)-C(4)	1.530(3)
O(8A)-C(6A)	1.351(3)
O(9A)-C(11A)	1.419(3)
O(10A)-C(12A)	1.419(3)
N(2A)-C(5A)	1.332(3)
N(2A)-C(9A)	1.338(3)
C(5A)-C(6A)	1.399(3)
C(5A)-C(10A)	1.489(4)
C(6A)-C(7A)	1.396(3)
C(7A)-C(8A)	1.411(3)
C(7A)-C(11A)	1.505(3)
C(8A)-C(9A)	1.383(3)
C(8A)-C(12A)	1.507(3)
O(8B)-C(6B)	1.352(3)
O(9B)-C(11B)	1.419(3)
O(10B)-C(12B)	1.422(3)
N(2B)-C(5B)	1.331(3)

N(2B)-C(9B)	1.344(3)
C(5B)-C(6B)	1.404(3)
C(5B)-C(10B)	1.487(4)
C(6B)-C(7B)	1.398(3)
C(7B)-C(8B)	1.403(3)
C(7B)-C(11B)	1.512(3)
C(8B)-C(9B)	1.379(3)
C(8B)-C(12B)	1.507(3)

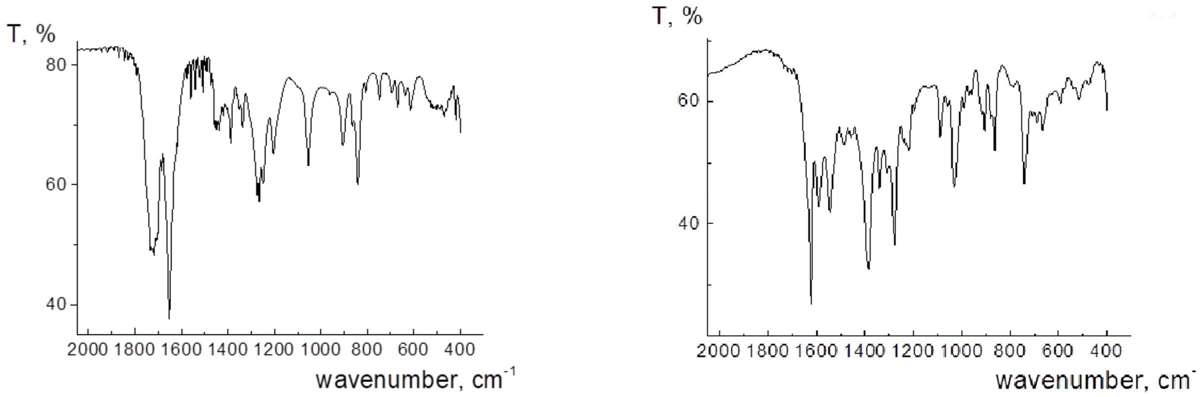

(**a**) (**b**)

Figure S2. (**a**) IR spectrum of the compound *I* (2-(nitrooxy)butanedioic acid); (**b**) IR spectrum of the compound *II* (di(3-hydroxy-4,5-bis(hydroxymethyl)-2-methylpyridinium) 2-(nitrooxy)butanedioate) (KBr pellet).

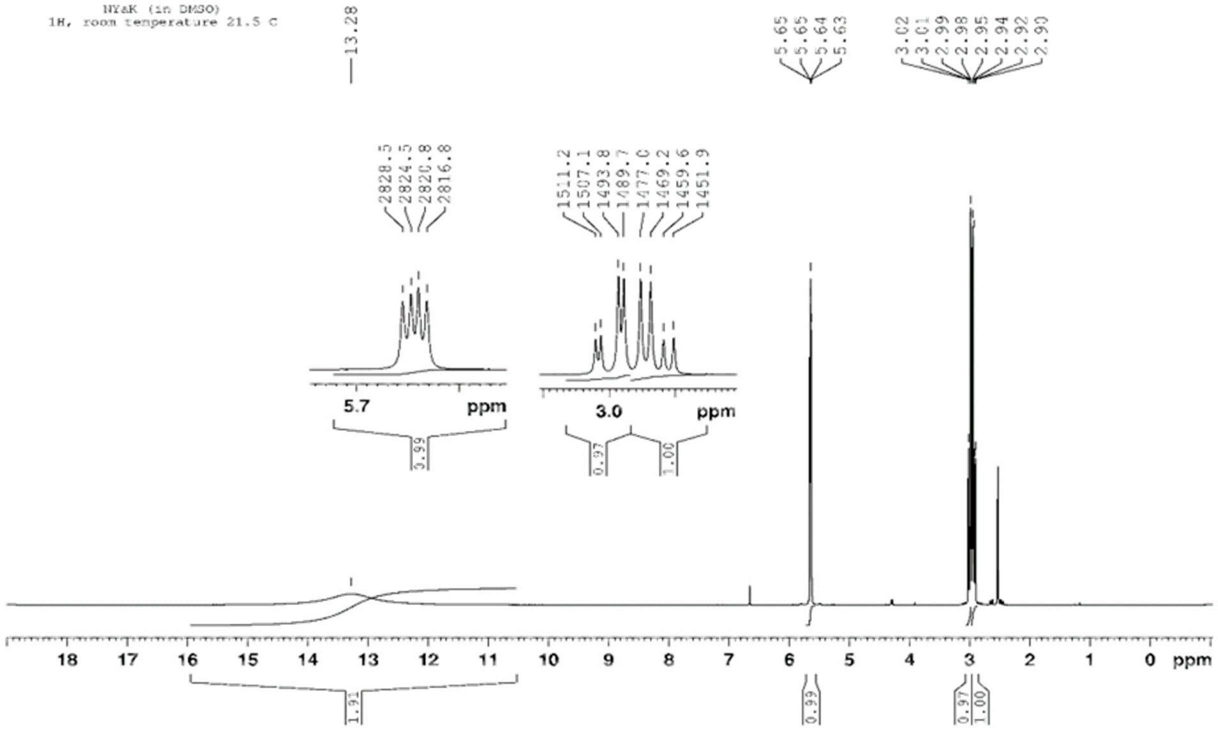

Figure S3. ^{1}H NMR spectrum of compound *I* (2-(nitrooxy)butanedioic acid) (DMSO-d6).

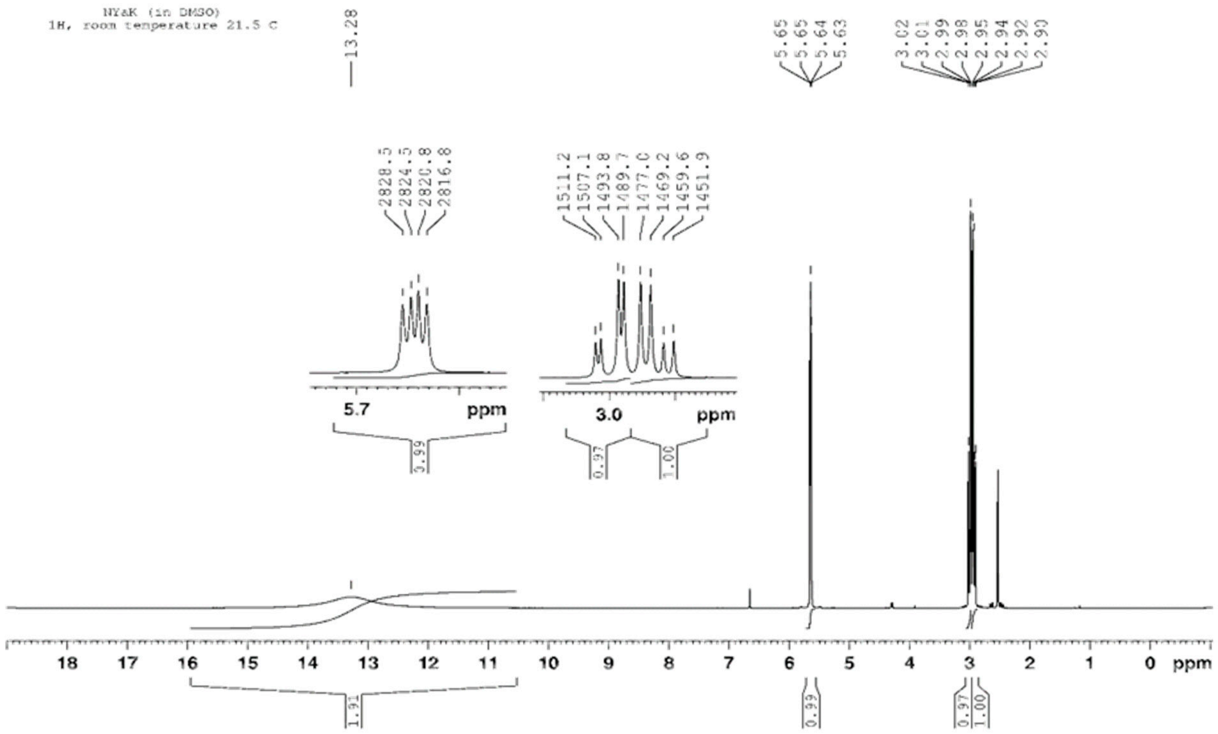

Figure S4. ^{1}H NMR spectrum of compound *II* (di(3-hydroxy-4,5-bis(hydroxymethyl)-2-methylpyridinium) 2-(nitrooxy)butanedioate) (DMSO-d6).

The expected characteristic signals of the carboxylate anion COO- appear in the IR spectrum of the target product *II*: 1620 cm^{-1} 1383 cm^{-1} and 743 cm^{-1} (Figure S2b). Also, when comparing the spectra of substances *I* and *II*, the disappearance of the signal in the region of 1650-1740 cm^{-1} is noticeable, which is characteristic of the vibration of H-linked C=O groups in dicarboxylic acids, which, in combination with the previous signals, allows us to assert about the complete conversion of acid *I* into the target product. In addition, the spectrum of compound *II* clearly shows double signals in the region of 830 - 930 cm^{-1} and 990 - 1100 cm^{-1}, which can be attributed to the vibration of C–O bonds in the phenolic groups of the pyridoxine residue of target product *II*. Thus, it can be argued that the IR spectrum is in complete agreement with the structure of compound *II*.

Comparison of the ^{1}H NMR spectra of *I* and *II* (Figure S3 and S4) also reveals the deprotonation of COOH-groups of acid *I* after the conversion to the salt form in compound *II* (chemical shifts ca 3.75 ppm in *I* and 13.28 ppm, respectively). As expected, signals of C-linked protons are not changed significantly.
